# Nematocytes: Discovery and characterization of a novel anculeate hemocyte in *Drosophila falleni* and *Drosophila phalerata*

**DOI:** 10.1371/journal.pone.0188133

**Published:** 2017-11-15

**Authors:** Julianna Bozler, Balint Z. Kacsoh, Giovanni Bosco

**Affiliations:** Department of Molecular and Systems Biology, Geisel School of Medicine at Dartmouth, Hanover, New Hampshire, United States of America; Institute of Plant Physiology and Ecology Shanghai Institutes for Biological Sciences, CHINA

## Abstract

Immune challenges, such as parasitism, can be so pervasive and deleterious that they constitute an existential threat to a species’ survival. In response to these ecological pressures, organisms have developed a wide array of novel behavioral, cellular, and molecular adaptations. Research into these immune defenses in model systems has resulted in a revolutionary understanding of evolution and functional biology. As the field has expanded beyond the limited number of model organisms our appreciation of evolutionary innovation and unique biology has widened as well. With this in mind, we have surveyed the hemolymph of several non-model species of Drosophila. Here we identify and describe a novel hemocyte, type-II nematocytes, found in larval stages of numerous Drosophila species. Examined in detail in *Drosophila falleni* and *Drosophila phalerata*, we find that these remarkable cells are distinct from previously described hemocytes due to their anucleate state (lacking a nucleus) and unusual morphology. Type-II nematocytes are long, narrow cells with spindle-like projections extending from a cell body with high densities of mitochondria and microtubules, and exhibit the ability to synthesize proteins. These properties are unexpected for enucleated cells, and together with our additional characterization, we demonstrate that these type-II nematocytes represent a biological novelty. Surprisingly, despite the absence of a nucleus, we observe through live cell imaging that these cells remain motile with a highly dynamic cellular shape. Furthermore, these cells demonstrate the ability to form multicellular structures, which we suggest may be a component of the innate immune response to macro-parasites. In addition, live cell imaging points to a large nucleated hemocyte, type-I nematocyte, as the progenitor cell, leading to enucleation through a budding or asymmetrical division process rather than nuclear ejection: This study is the first to report such a process of enucleation. Here we describe these cells in detail for the first time and examine their evolutionary history in Drosophila.

## Introduction

The innate immune system of animals is an essential element that protects against environmental insults and increases one’s survival. Examination of the innate immune system has revealed conserved signaling from fruit flies to humans, providing insight into an organism’s ability to rapidly adapt to the environment. Towards this end, many species have been studied to uncover fundamental principles of the innate immune system. *Drosophila melanogaster* has been one such model for the investigation into innate immunity; the myriad of molecular genetic tools in this system has heavily focused studies of insect immunity on this one species. *D*. *melanogaster* has three immune cell types; plasmatocytes, lamellocytes, and crystal cells [1]. Plasmatocytes are phagocytic cells responsible for removal of bacterial and fungal infections. These cells comprise the bulk of hemocytes in the unchallenged *D*. *melanogaster* larvae, an observation that holds true for other Drosophila species [2,3]. Crystal cells are generally involved in wound healing and melanization [4,5]. Finally, lamellocytes are specialized immune cells responsible for containment and removal of macro-infections, such as parasitic wasp eggs, through the formation of cellular capsules [6]. This immune response is functionally akin to granuloma formation in humans, although cell types responsible for these processes do not appear to be homologous [7]. Similarities such as these point to convergent adaptations in response to universal pressures and demands on the immune system. This raises the likely possibility that other functionally convergent or entirely novel aspects of innate immunity remain to be discovered in other organisms. Continuing to uncover new immune cells and processes can provide useful insight into both conserved and unique molecular adaptions to shared environmental threats.

The primary immune cell types described in *D*. *melanogaster* are not the only immune effectors present in more distantly related Drosophila species. In addition to plasmatocytes, *Drosophila willistoni* have an unusual cell type termed nematocytes [8]. Nematocytes were initially studied observationally and characterized by their long spindles projecting from an oblong cell body. More recent work on nematocytes in the Drosophilid *Zaprionus indianus* has found the cells to be involved in non-melanotic encapsulation of macro-infections [9].

With the intent to explore some of these novel immune processes, we chose to examine the immune system of less studied Drosophila species. We find that the mushroom specialist Drosophilids, *Drosophila falleni* and *Drosophila phalerata*, also have nematocytes. Further investigation into this cell type revealed an additional, previously undescribed cell, morphologically similar to the classical nematocyte. We term this cell a type-II nematocyte; interestingly, this new class of nematocyte is enucleated, but capable of movement and protein synthesis. These remarkable cells are able to form complex multi-cellular webbed structures. Here we provide a detailed characterization of these cells, and propose that these anucleate nematocytes help facilitate the multicellular immune structures generated in response to macro-infections.

## Methods

### Fly husbandry

Flies were maintained at room temperature on standard Drosophila cornmeal-molasses media; refer to S1 Table for stock listing and source information. Food media for *Drosophila falleni* and *Drosophila phalerata* was supplemented with yeast extract and mushrooms. *Drosophila palustris* media was supplemented with yeast extract and banana. Third instar larvae were collected from 5-day egg-lays with approximately 150 female flies in a 15 x 9 cm chamber.

### Immunofluorescence

Late wandering stage larvae were used for all experiments. For imaging purposes, hemolymph from five larvae in 20 μl PBS was placed on a poly-l-lysine treated coverslip and allowed to incubate for 30 minutes. Cells were then fixed in 4% paraformaldehyde for 10 minutes and prepared for staining.

Samples for immunofluorescence were blocked with 2% NGS in PBS for one hour. Primary antibodies of rat anti-tubulin (YL1/2), and mouse anti-nuclear pore complex (Abcam Mab414) were used at concentrations of 1:500 and 1:2000 respectively. Samples were incubated in primary antibody solution overnight at 4°C. At the conclusion of the incubation, samples were washed, blocked, and incubated with secondary antibody of FITC or Cy3 conjugates (Jackson Immunoresearch), used at a concentration of 1:150. Samples were counter stained with 4’, 6-diamidino-2-phenylindole (DAPI) (1 μM).

### Cell stains

Propidium iodide (PI) staining was used to detect nucleic acid and cell viability. Following fixation (as described above), nucleic acid staining was performed by incubating the samples in PI (1 μM) in PBS for 30 minutes. Samples were counter stained with DAPI and a no point were treated with RNase. Alternatively, for cell viability, cells were incubated with the PI solution prior to fixation.

The membrane marker wheat germ agglutinin (WGA) conjugated with Alexa Flour 488 (Life technologies) was used as an additional cell marker. Post-fixation, cells were incubated in a 10μg per mL solution for 30 minutes.

Protein synthesis was measured with Click-it HPG Alexa Fluor 488 protein synthesis assay (Thermo Fisher, C10428) according to manufacturer’s instructions. As a negative control, cells were treated with cycloheximide (50μg/mL) during the protein synthesis incubation.

Fluorescent detection of mitochondria was conducted with MitoTracker Red (Thermo Fisher, M7512). Staining was performed on live cells and fixed for imaging, per the manufacturer’s instructions.

Phagocytosis assay used green fluorescent beads 1 micron in size, and were a generous gift from the Berwin Lab (Dartmouth College). Hemolymph from 5 larvae was incubated with 1 μl of beads for 1 hour prior to imaging.

### Fluorescent microscopy

Confocal images were captured with the Nikon AIR S1 confocal microscope and Nikon Elements imaging software. Image averaging of 4x was used during image capture. Standard fluorescent images were visualized with the Nikon Eclipse E800 microscope and the Olympus DP71 camera. Pertaining to tubulin images collected for the phylogenetic tree exclusively, images were taken at multiple focal planes and merged to capture the complete cell; contrast and brightness were adjusted in Photoshop to clarify structural features. The type of microscopy used for particular experiments is indicated in figure legends.

### Colchicine treatment

Cells were treated with 0.4mg/mL colchicine or untreated and incubated for 30-minute on a poly-L-lysine coverslip. Samples were then fixed and stained with WGA and DAPI. Standard fluorescent microscopy was used for analysis. Given the difficulties of determining the number of cells within a cluster or web-like structure, the analysis was based on size only. Of all three replicates in the colchicine treatment, no webs of type-II nematocytes were observed. Therefore, quantification focused on type-I nematocytes. Cells with a length less than twice the diameter of its nucleus were classified as not having standard spindle projections.

### Electron microscopy

Samples for SEM were prepared using previously described methods [10]. Briefly, hemolymph from 5 larvae were incubated in PBS on a coverslip for 30 minutes. Fixation with 1% GTA in 0.1M NaCacodylate was incubated for 15 minutes at room temperature before transfer to 4° for overnight fixation. Fixative was rinsed and dehydrated in serial dilutions of ethanol. Samples underwent critical point drying and Osmium coating (6nm).

Samples for TEM were prepared using previously described methods, with the exception of sample mounting [11]. Hemocytes were allowed to settle on glass coverslip, and fixed with a 2% glutaraldehyde and 1% paraformaldehyde solution in 0.1 M NaCacodylate. After processing, samples were embedded with the glass coverslip, after embedding coverslip was subsequently etched away so that cells could be viewed with intact morphology. Samples were then sectioned and stained. Microscopes used for these experiments were the FEI XL-30 ESEM-FEG scanning electron microscope and the JEOL JEM 101 transmission electron microscope.

### Live cell imaging

Live cell DIC images were captured using the inverted microscope Nikon Eclipse Ti with Nikon Elements software. Cells were maintained in PBS on a poly-L-lysine coverslip mounted on an aluminum plate. Images were taken at 2-minute increments with Nikon’s perfect focus and 60x objective. Phase contrast imaging relied on the upright Nikon Eclipse E800 microscope. Images were taken with a 100x oil emersion objective.

## Results

### Hemocyte composition

**Fig 1:**
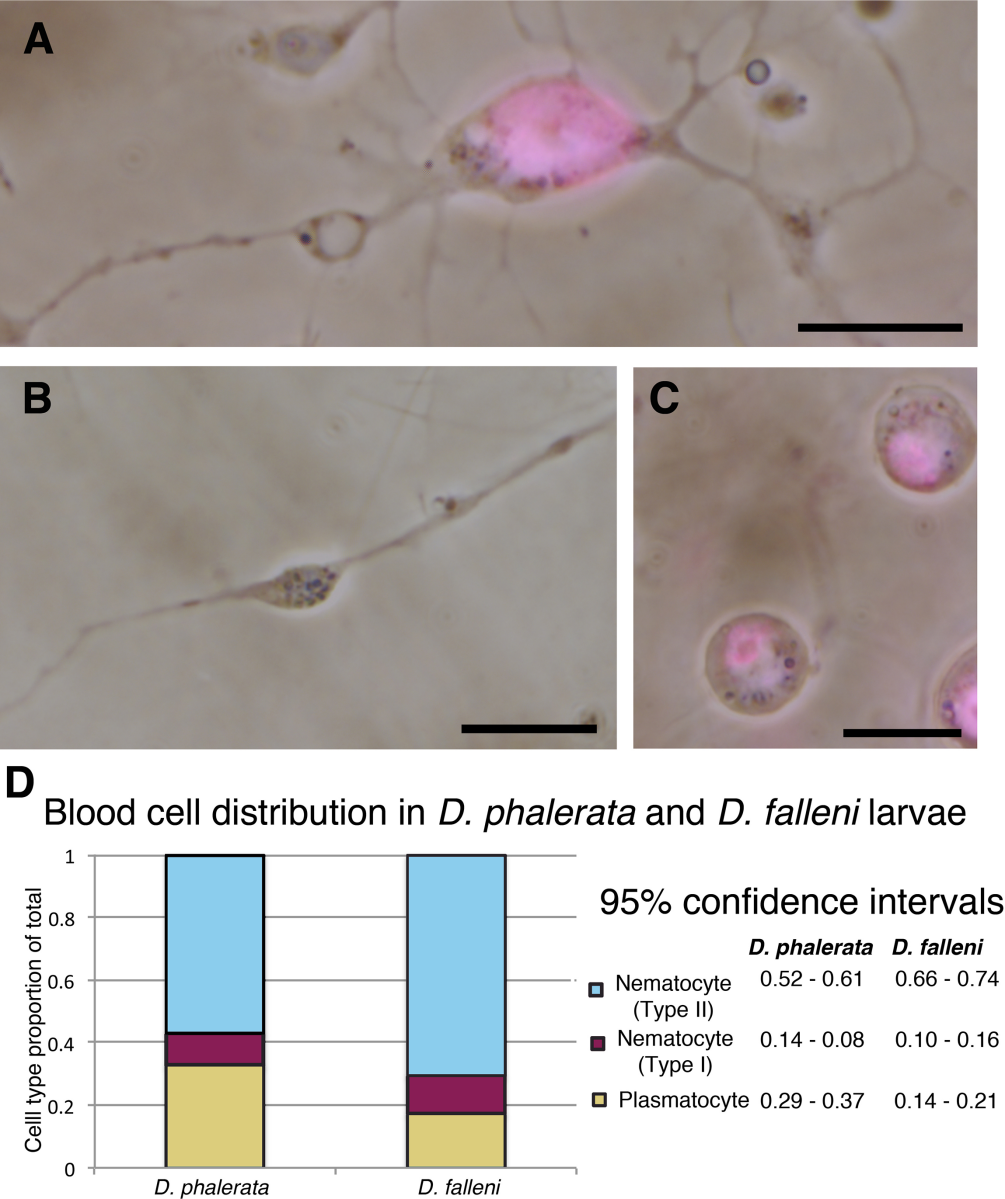
Three distinct hemocytes are found in 3^rd^ instar larvae of *Drosophila phalerata* and *Drosophila falleni*. Live cell imaging of three cell types, counter stained with DAPI in magenta, shows two classes of nematocytes. Nucleated nematocyte (type I) with strong DAPI signal and spindle projections extruding from the cell body (**A**), and enucleated nematocyte (type II) lacking DAPI staining but morphologically similar, having spindle projections extending from central cellular region (**B**). DAPI positive plasmatocytes are also found in circulating hemolymph (**C**). The distribution of hemocyte populations in each species was quantified (**D**), with type II nematocytes being the most abundant cell type in both species. Scale bars are 10 microns (**A**-**C**).

We examined hemolymph from third instar larvae of the mushroom specialists *Drosophila falleni* and *Drosophila phalerata*, and identified three cell types with distinct morphologies. Two cell types were DAPI positive in unfixed samples, indicating the presence of DNA (Fig 1A–1C). These cells fit the typical morphology of previously described cells found in other Drosophila species, termed plasmatocytes and nematocytes. The third cell type was unique in that it was DAPI negative. Abundance of these cell types were quantified based on physical traits observable with light microscopy (Fig 1D). Specifically, plasmatocytes are small round phagocytic cells that have a noticeable nucleus. There are then two classes of nematocytes: Type-I, contain a large nucleus that occupies the bulk of the cell body and have long projections extruding from the cell body. Type-II nematocytes tend to have a central bulge within the cell from which narrow spindles extend. Most notably these cells lack detectable DNA, this anucleate status (lacking a nucleus) is one of the primary distinguishing features of this cell type. Quantification revealed that type-II nematocytes were the most abundant cell type in both species, approximately 56.7% in *D*. *phalerata*, and 70.3% in *D*. *falleni*. Plasmatocytes constituted the majority of the remaining cells, 32.8% in *D*. *phalerata*, and 17.3% in *D*. *falleni*. Whereas type-I nematocytes were the least frequent, 10.5% and 12.4% for *D*. *phalerata* and *D*. *falleni* respectively.

### Characterization and structure of nematocytes

Based on these initial results, we chose to further characterize this novel anucleate nematocyte class. Although neither species is a model system, certain generalizable stains and antibodies are still available for their study. An antibody to a conserved region of the nuclear pore complex was used to assay for the presence of a functional nuclear membrane. No such structure was detected in type-II nematocytes, although both plasmatocytes and type-I nematocytes showed clear nuclear pore staining (S1A–S1C Fig). This result validates the previous finding, through DNA staining, that type-II nematocytes lack a defined nucleus.

**Fig 2:**
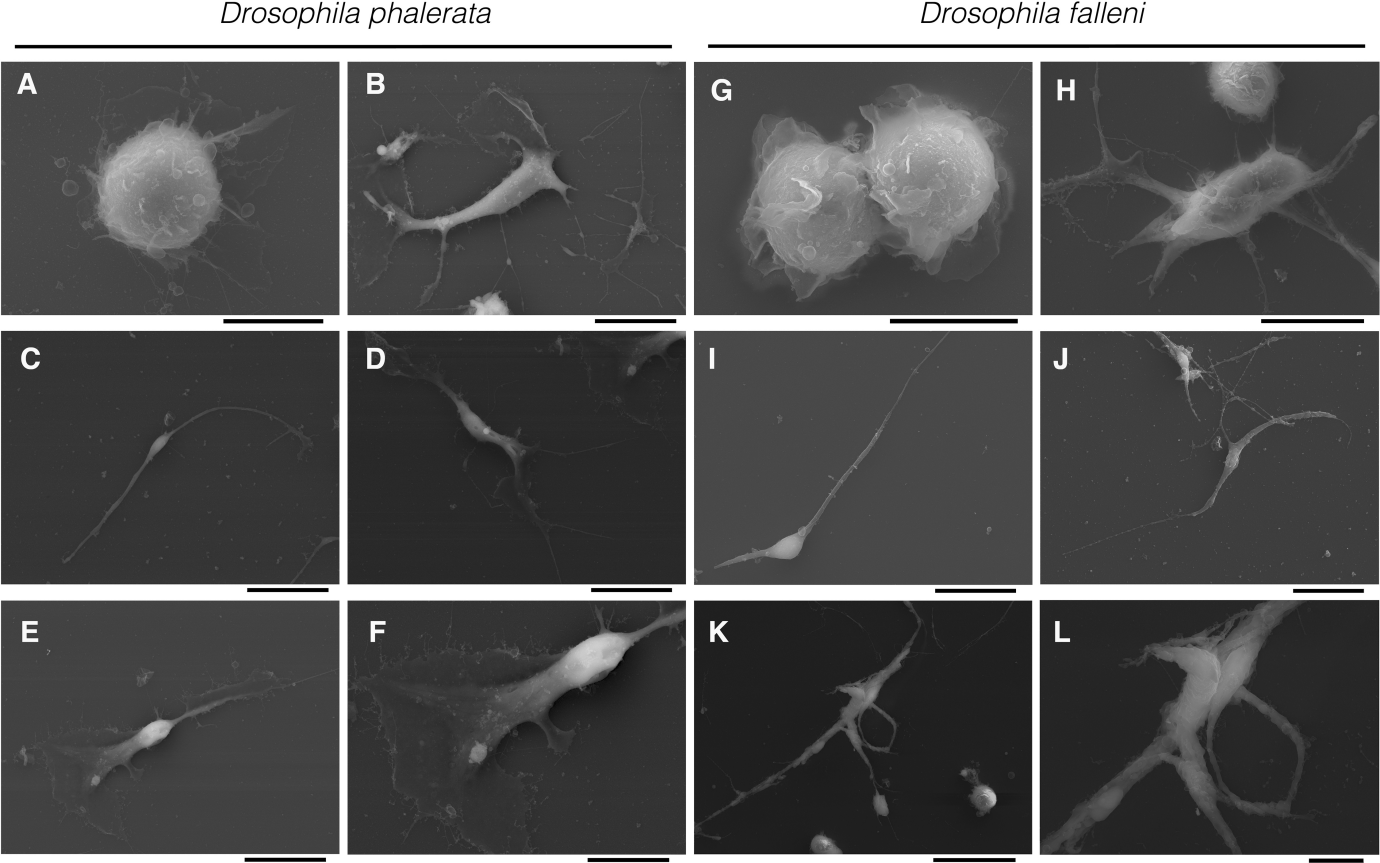
Hemocytes in *Drosophila phalerata* and *Drosophila falleni* examined with scanning electron microscope. Three cell types in *D*. *phalerata* are seen including plasmatocytes (**A** [5 μm]), type I nematocyte (**B** [10 μm]), and type II nematocytes (**C** [5 μm], **D** [10 μm], **E** [10 μm]). Magnification of panel E shows cell spreading in type II nematocyte (**F** [5 μm]). The same cell types in *D*. *falleni* are observed, including plasmatocytes (**G** [5 μm]). Type I nematocyte is shown with the characteristic projections extending from the cell body (**H** [10 μm]). Morphologically distinct type II nematocytes are shown with typical narrow spindles (**I** [10 μm], **J** [10 μm]). Final panels illustrate two type II nematocytes interacting (**K** [20 μm]) along with magnification of the interaction point (**L** [5 μm]). Scale bars are indicated in brackets.

**Fig 3:**
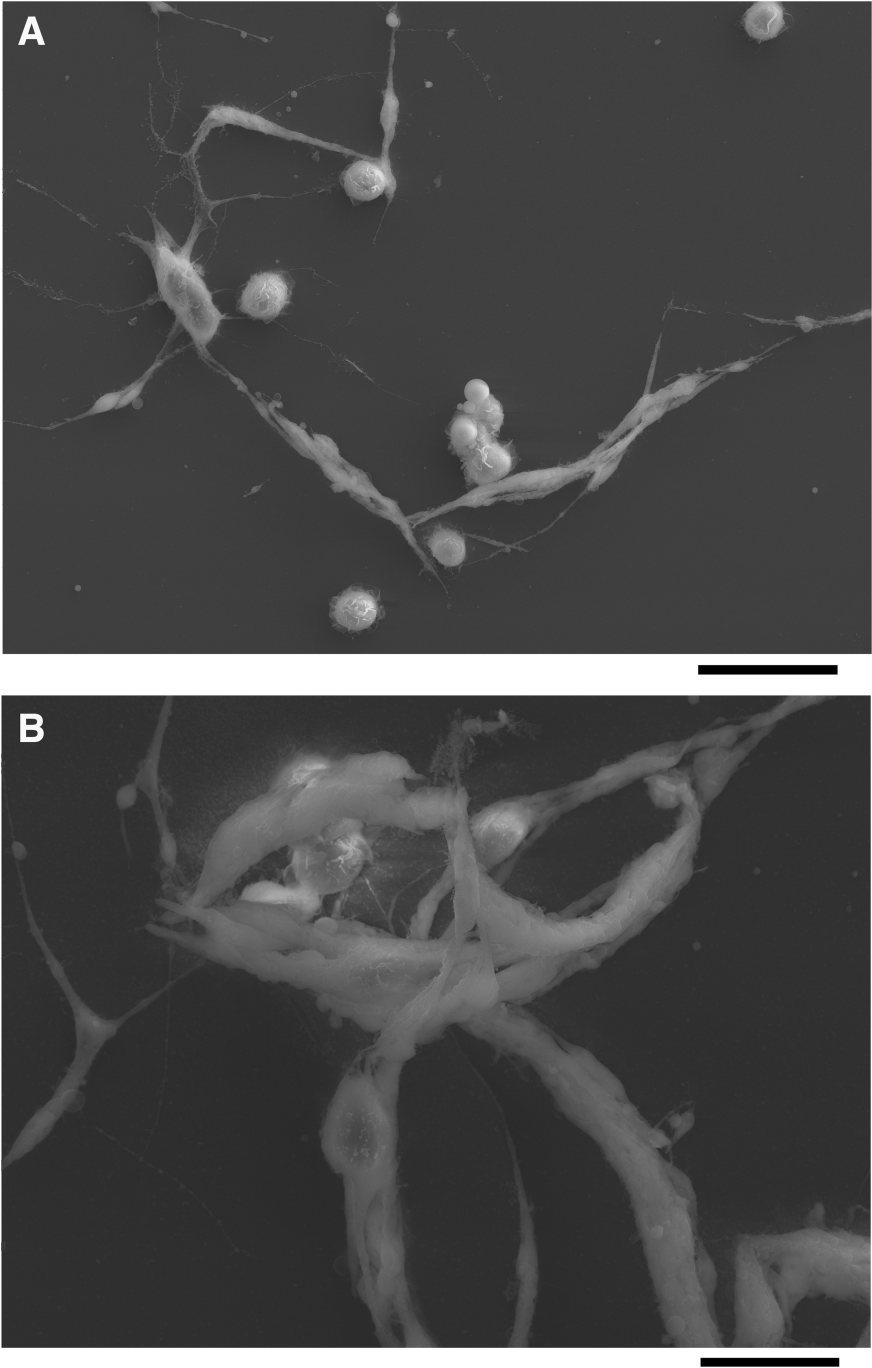
Nematocytes form multicellular structures, observed with scanning electron microscope. Distinct nematocytes (type I & type II) from *D*. *Falleni* are seen intertwined, forming large, web-like structures (**A**, **B**). Scale bars are 20 microns and 10 microns respectively.

Scanning electron microscopy (SEM) revealed similar morphologies to those observed with standard phase contrast light microscopy. Again, each of the three cell types was observed in *D*. *phalerata* and *D*. *falleni* (Fig 2, S2 and S3 Figs). Further, nematocytes, appeared to connect or intertwine with other cells, forming multicellular structures (Fig 3). These structures range in size from only two nematocytes (Fig 2L), to many cells. Further, these large webbed structures can include only type-II nematocytes, or be a mix of type-I and type-II nematocytes (Fig 3).

Propidium iodide (PI) is considered a vital stain, meaning that it is not permeable to live cells. To assess the viability of these cell types, unfixed hemolymph samples were stained with PI. Type-I and type-II nematocytes were negative for PI fluorescence, demonstrating that these cells are alive and have intact membrane (S4 Fig).

PI fluoresces when it is bound to either DNA or RNA, using the same PI assay in paraformaldehyde fixed cell, and performed in parallel with the live cell vital staining, we examined the presence or absence of nucleic acids in these cell types. Fluorescence from the PI was clearly visible in both type-I and type-II nematocytes (S4 Fig). However, no DAPI signal was observed in type-II nematocytes; indicating that DNA negative cells contain RNA molecules. Taken together these data indicate that type II nematocytes are functional cells that lack a nucleus, but unexpectedly, do have RNA.

**Fig 4:**
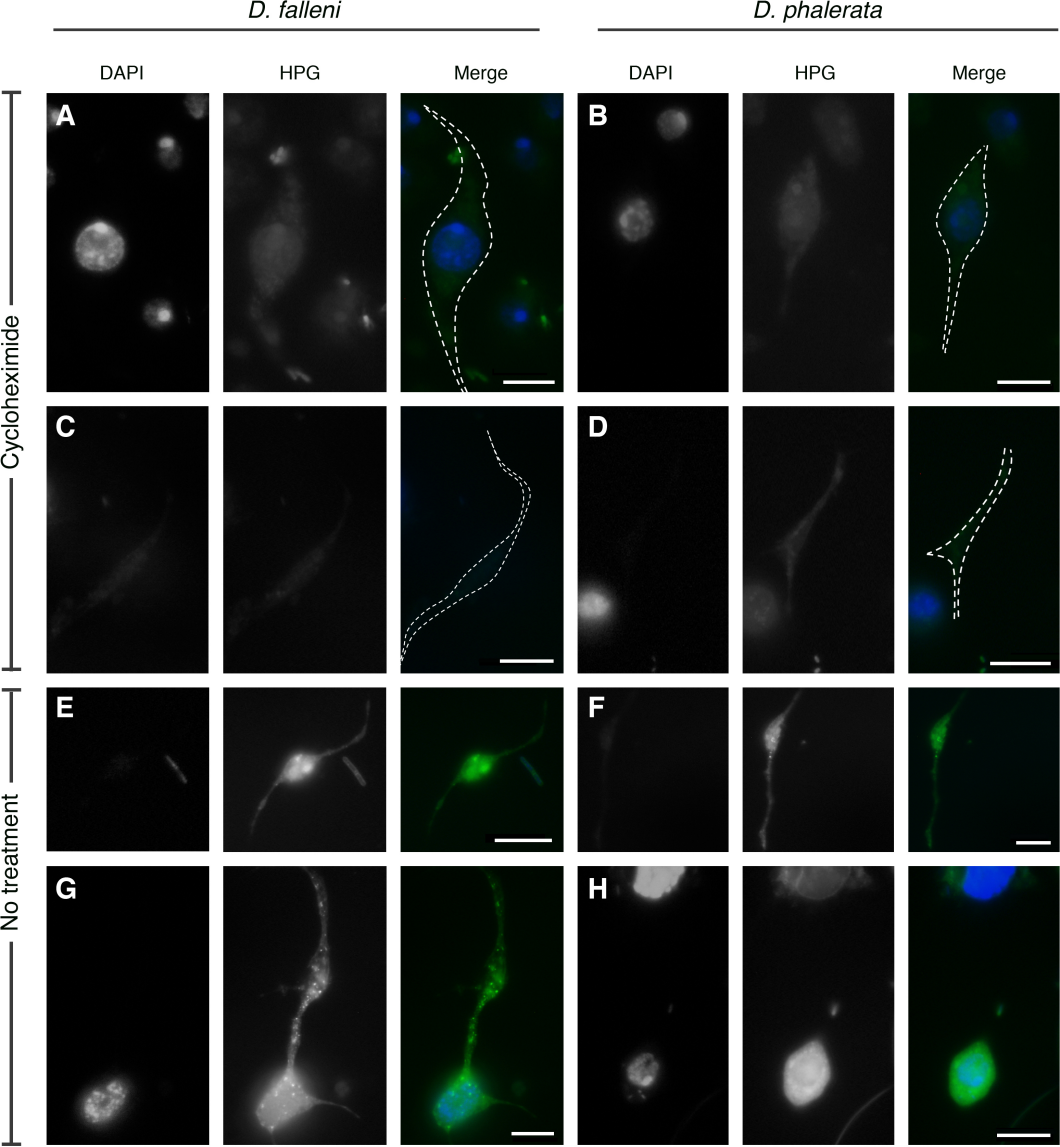
Hemocytes are able to synthesize protein. Nascent protein synthesis was visualized using the Click-it protein synthesis kit, measured with green fluorescence (HPG). DNA is marked with DAPI staining. As expected, type-I nematocytes (A & B) and type-II nematocytes (C & D) in the cycloheximide treatment did not indicate significant levels of protein synthesis. However, these cells without cycloheximide treatment had green fluorescence, signifying protein synthesis. White dashed line in merged images denotes the cell outline as based on phase-contrast image. Images were taken at identical microscope and camera settings with standard fluorescent microscope. Scale bars are 10 microns.

The presence of RNA is uncommon to many enucleated cells and raised questions about cellular functions, such as the ability to synthesize proteins. We used a fluorescently based protein synthesis assay to explore this possibility: This assay measures the incorporation of a methionine analog into nascent proteins. Fluorescence was detected in plasmatocytes, type-I nematocytes, and type-II nematocytes, indicative of ongoing protein synthesis in all hemocyte types. Importantly, this fluoresces was not detected when protein synthesis was blocked with the addition of cycloheximide to the media (Fig 4).

**Fig 5:**
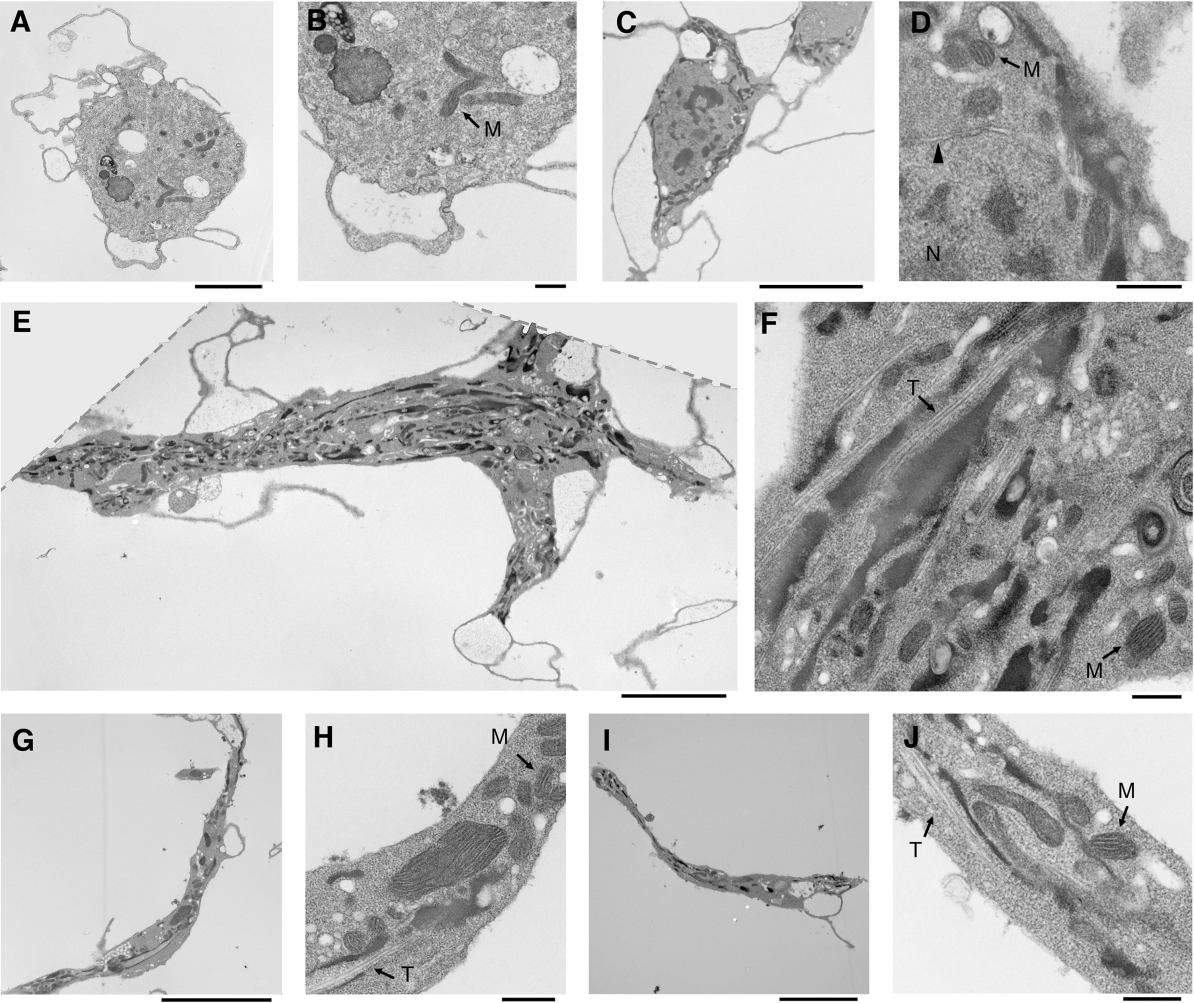
Cellular structure and organelle composition of *Drosophila phalerata* hemocytes visualized with transmission electron microscopy. Ultrastructure and organelles of three hemocyte classes were captured; key features include nucleus (N), nuclear envelope (carrot), microtubules (T), and mitochondria (M). Based on morphology, imaged cells are classified as plasmatocytes (**A** [2 μm]) and corresponding magnification (**B** [500 nm]), a type-I nematocyte (**C** [5 μm]) and magnification (**D** [500 nm]). Finally, type-II nematocytes are shown, and clearly possess long microtubules and mitochondria (**E** [5 μm], **G** [5 μm], & **I** [5 μm]), respective magnifications are also shown (**F** [500 nm], **H** [500 nm], & **J** [500 nm]). Scale bar lengths are indicated within brackets.

Further investigation into the structure and composition of type-I and type-II nematocytes was conducted using transmission electron microscopy (TEM) (Fig 5, S5 and S6 Figs). DNA and nuclear envelope are noted in plasmatocytes and type-I nematocytes. Microtubule filaments were highly profuse in both nematocyte classes. Interestingly, although mitochondria were observed in each of the three cell classes, type-II nematocytes had a surprising abundance.

We suspected a significant role of cytoskeletal filaments in nematocytes, given their unusual morphology and long narrow protrusions; therefore, the high abundance of microtubules was not surprising. Confirmation of this observation was performed with tubulin antibody staining, which revealed significant microtubule networks in both type-I and type-II nematocytes (S7 Fig). A final organelle assay tested the presence of mitochondrial: All three cell types were positive for mitochondria based on MitoTracker fluorescence, a dye active based on mitochondrial membrane potential (S1D–S1F Fig). These additional microscopy techniques validate the general structural findings of the TEM imaging.

### Functional characterization

**Fig 6:**
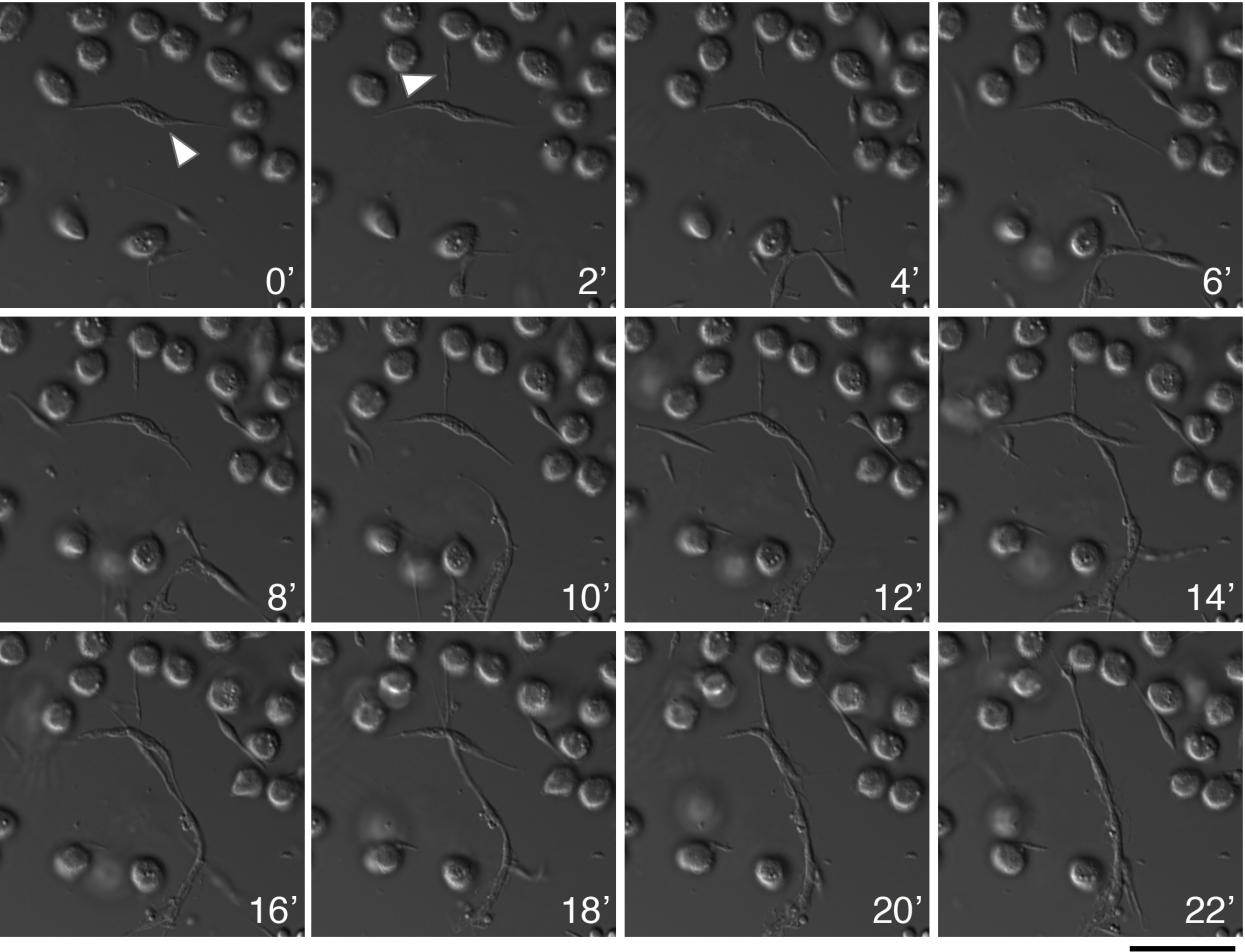
Type-II nematocytes are dynamic, mobile, and capable of interacting with other cells. Time-lapse imaging shows two type-II nematocytes (T0 & T2) dynamically growing and shrinking. By the final time point these cells have intertwined or connected to neighboring cells, forming a multicellular structure. Time intervals are 2 minutes with DIC microscopy and perfect focus. White carrot indicates cells of interest. Scale bar is 20 microns.

The distinct and complex composition of type-II nematocytes points to a functional role within the late-stage larvae. A phagocytosis assay with fluorescent beads revealed no detectable phagocytic activity in type-II nematocytes, although plasmatocytes did show engulfment of beads (S8 Fig). An alternative hypothesis is that these cells participate in the macro-parasite immune response. With live cell imaging, we were able to observe that type-I and type-II nematocytes are highly dynamic and mobile. Further, this imaging revealed the type-II nematocyte’s ability to actively link to other cells, forming large multicellular web-like structures (Fig 6, S1 and S2 Media Files). It is possible that these massive complexes are a part of the non-melanotic encapsulation immune process for isolating macro-parasites. With the high abundance of microtubules in both type-I and type-II nematocytes, it is reasonable to speculate that tubulin dynamics are partly responsible for the large and multicellular structures. Indeed, when microtubule assembly is inhibited with colchicine, type-I nematocytes and their multicellular structures were greatly limited in size (S9 Fig), no type-II nematocytes or related structures were detected. Such findings may indicate that type-II nematocyte formation requires microtubules, or that microtubules are necessary for the cell’s stability; although not mutually exclusive. In many instances type-I nematocytes had no spindle projections and were only recognizable by their large uniquely structured nucleus, lending some credibility to the latter hypothesis. Plasmatocytes were generally unaffected.

**Fig 7:**
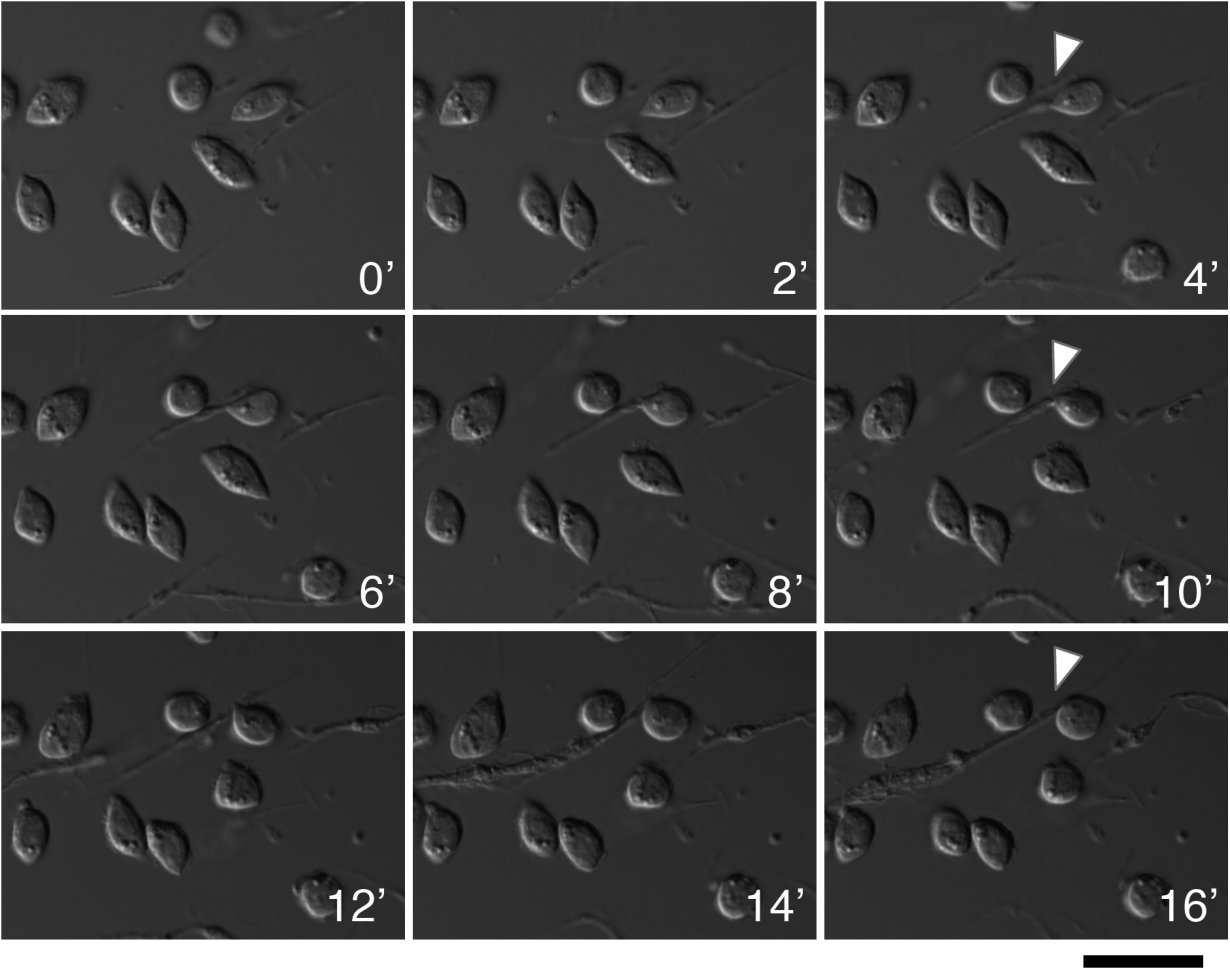
Live imaging captures formation of type-II nematocyte. Time-lapse imaging shows the formation of a spindle-like projection and an eventual pinching off of this appendage from the progenitor cell (T12). This budded cell maintains dynamic movement after separation. Time intervals are 2 minutes with DIC microscopy and perfect focus. White carrot indicates specific region of interest. Scale bar is 20 microns.

Live-cell imaging experiments showed additional dynamics of the type-I and type-II nematocyte relationship. It is notable that many of the type-II nematocytes structurally resemble type-I nematocytes, yet lack a nucleus and are often times smaller in size. We observed on several occasions the pinching off of a projection from a type-I nematocyte, which we propose to be the creation of a type-II nematocyte (Fig 7 and S3 Media File).

### Phylogenetic analysis of nematocytes

**Fig 8:**
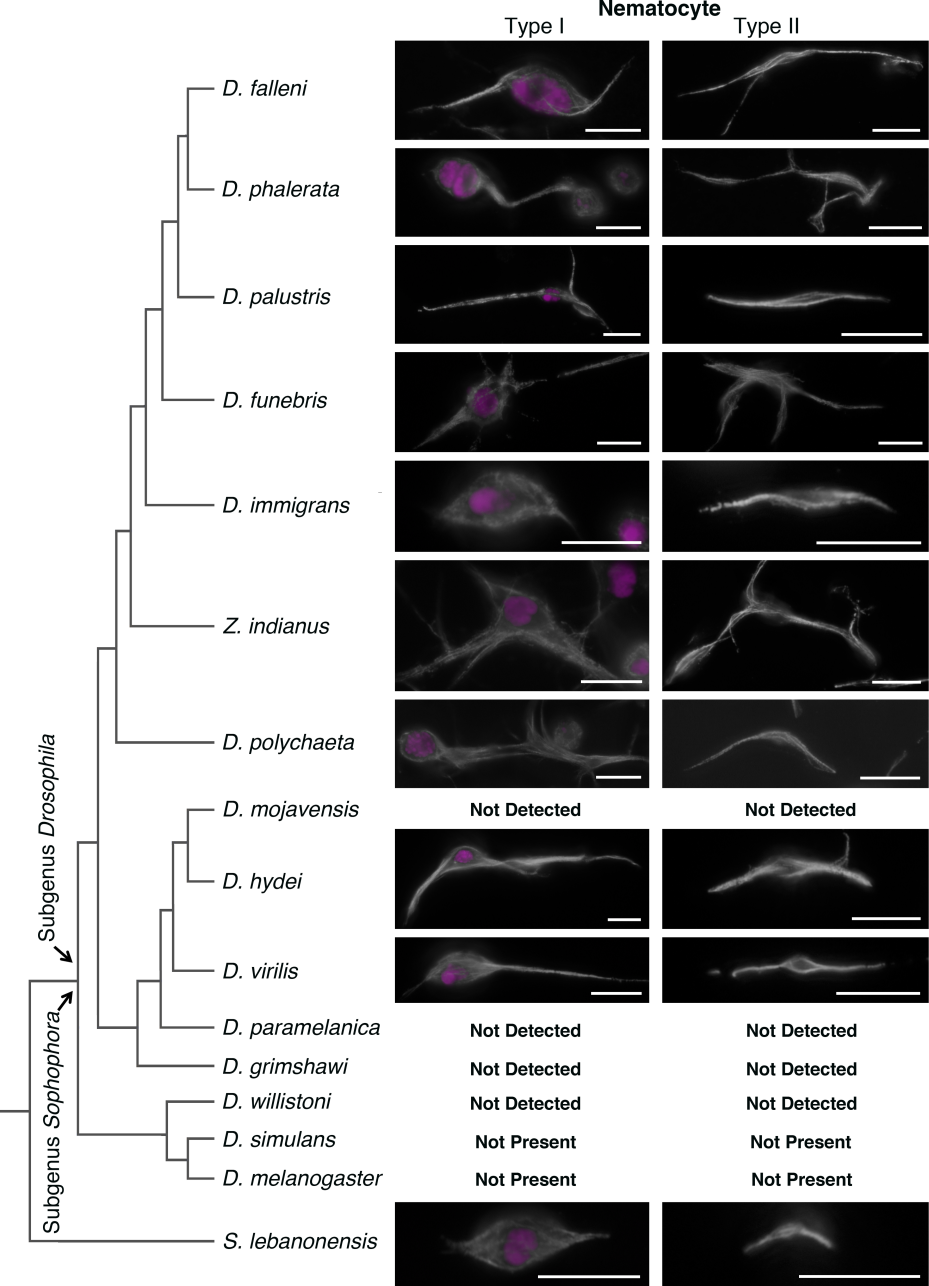
Numerous Drosophila species have type-I and type-II nematocytes. Hemolymph from multiple Drosophila species was examined for the presences of type-I and type-II nematocytes. Images show tubulin (white) and DAPI (magenta). “Not Detected” reports that the cell type was not detected from multiples samples in this study. In *D*. *melanogaster* and *D*. *simulans* these cell types are assigned a “Not Present” status, reflecting that these cell types are not present based both on the current findings and supported by a significant body of literature. The left panel reports a phylogenetic tree for these species; however, horizontal length of the branches does not represent a quantified genetic distance or evolutionary time. Scale bars are 10 microns.

Nematocytes have been described in several species previously. However, a careful examination with the morphological characteristics defined in this paper has not been performed. Therefore, we collected hemolymph from a number of Drosophila species, and characterized blood cells with anti-tubulin and DNA (DAPI) staining. We found that type-I and type-II nematocytes are commonly found in a wide range of Drosophilids, although not in the melanogaster subgroup (Fig 8). It is important to note that neither type of nematocyte was detected in *D*. *willistoni* or *D*. *grimshawi*, despite previous reports [8,9]. This discrepancy may be due to strain-specific differences or fly-rearing conditions, amongst other possibilities. Although *D*. *grimshawi* did not have nematocytes as we have classified them, large multicellular, and multinuclear structures were observed; yet they lacked the distinctive microtubule spindle projections that we associate with both types of nematocyte. In all species that were found to be nematocyte-positive, both type-I and type-II nematocytes were observed. On no occasion was one type of nematocyte found without the other.

## Discussion

Innate immunity is a powerful component of the mammalian immune system. However, Drosophila, like most insects, lack any such adaptive immunity and are therefore fully dependent on innate immunity processes [12,13]. Studying the immune defenses developed by various insects has revealed novel cell types as well as provided insight into specific ecological challenges faced by these organisms [14,15]. One such pressure on many Drosophilids is the persistent threat of endoparasitoid wasp infection [16,17]. Drosophila melanogaster larvae respond to these macroparasites by mounting a cellular immune response, resulting in a multi-cellular capsule forming around the wasp egg; encasing the parasite and isolating it from the rest of the larva’s body [5,18].

Other drosophila species have differing immune response to this threat: Evolution across the genus has resulted in a variety of chemical and cellular defense responses to wasp infection [19,20]. *Zaprionus indianus* for instance has a cellular encapsulating defense involving nematocytes, a hemocyte not observed in D. melanogaster [9]. In this study, we report that the mushroom specialists *Drosophila phalerata* and *Drosophila falleni* constitutively express nematocytes in the late larval stages. Further investigation of these cells revealed two distinct cell types within the nematocyte class. Type-I nematocytes are consistent with previous descriptions of nematocytes, and contain typical cellular features including nucleus, RNA, mitochondria, and an extensive microtubule network as shown through fluorescent microscopy and TEM. These cells have unique and specific morphological characteristics, easily identified by their long, narrow arms extending from the cell body. Similar in gross morphology are type-II nematocytes. These cells tend to be smaller in size, but still have long spindle arms typically projecting from a cellular bulge. Importantly, these cells lack a nucleus, making them distinct from all previously described drosophila hemocytes.

When exploring biological novelties, structural properties of cells can be suggestive of their functional role. One distinctive and telling characteristic is the presences or absence of a nucleus. Anucleate cells (those lacking a nucleus) are rare, but have evolved independently in multiples species [21]. Humans have the best-known example of such cells: Erythrocytes, or red blood cells undergo a process of enucleation where the cell ejects its nucleus during the final stages of maturation [22,23]. In a case of convergent evolution, salamanders also have enucleated blood cells, again used for oxygen transport rather than immune defenses [24]. By most observations, cells without DNA typically have limited protein synthesis capacity and are therefore traditionally considered short lived and replaceable. However, this is not always the case, as one study described enucleated neurons in the micro-wasp *Megaphragma mymaripenne*, a phenomenon achieved through nuclear lysis rather than ejection [25]. It is speculated that spatial constraints on this animal are responsible for this innovative adaptation [24,25]. Understanding these biological peculiarities may provide insight into cell survival strategies.

Until recently, enucleated cells had been considered limited in lifespan and cellular processes [26,27]. However, type-II nematocytes have many shared features with the nucleated type-I nematocytes; both can synthesize protein, dynamically modify their shape, and form multicellular structures. These are unexpected characteristics for cells without a nucleus and may present novel biological processes. Immunofluorescence and TEM revealed a significant network of microtubules in these cells. In addition, live cell imaging captured these cells’ unusual ability to extend and retract their spindle appendages, sometimes even joining or connecting to adjacent cells. Furthermore, based upon experiments with a tubulin assembly inhibitor we propose that this dynamic movement and structural formations are microtubule dependent processes. Given these findings, we speculate that type-II nematocytes facilitate the formation of multicellular immune structures, similar to those seen in *Zaprionus indianus*.

In previously described cells, enucleation occurs through the ejection of the nucleus from the differentiating cell, or in one curious case, the selective lysis of the nucleus [22,25,28]. Here we offer evidence for an additional process of enucleation, where asymmetrical division, or budding, of the anucleate cell from the body of the nucleated progenitor results in a cell devoid of a nucleus. Live cell imaging captured a type-I nematocyte developing a spindle appendage and releasing it from the rest of the cell body. We propose that this is the process through which type-II nematocytes form.

The possibility existed that these type-II nematocytes were a biological aberration restricted to the mushroom specialists. Yet, close examination of other drosophila species exposed the presence of both type-I and type-II nematocytes across a range of Drosophila species, indicating that perhaps the well-studied melanogaster subgroup is a phylogenetic outlier in this respect. It is interesting to note that in nematocyte-positive species, both type-I and type-II nematocytes were observed. In no instance was one type of nematocyte found without the other; which at a minimum serves as a suggestive coincidence further pointing to a cellular relationship, functional or otherwise.

This study presents the first example of anucleate hemocytes in insects. Although the linage of type-I nematocytes is unclear, originating from a specialized organ or differentiating from plasmatocytes: It nonetheless appears through live cell imaging, that type-II nematocytes originate from the larger type-I nematocyte. Further investigation into these novel cells will provide insight into the signaling and induction leading to this asymmetrical cell budding and enucleation. Understanding the clear mechanisms behind the generation of these anucleate cells may illustrate novel and generalizable biological processes while also providing insight into the evolution of insect immunity. It is tempting to speculate that similar anucleated cells exist and function in the innate immune system of other animals. This study provides a foundation for future research with a thorough initial description of these blood cells and their evolutionary heritage across the genus drosophila.

## Supporting information

S1 Fig Type-I and type-II nematocytes imaged for nuclear pores and mitochondria. Nuclear pore complexes were observed in both DAPI positive cell types; plasmatocytes (**A**) and type-II nematocytes (**B**), as detected by immunofluorescence. Type-II nematocytes are negative for both DAPI and nuclear pore complex signal, making them distinct from the other two hemocytes (**C**). Mitochondria were detected using MitoTracker-Red and pseudo colored green. Each of the cell types; plasmatocytes (**D**), type-I nematocytes (**E**), and type-II nematocytes (**F**) are positive for this stain. DAPI is shown in magenta within the merged panels and the cell outline (white dashed line) is based on the respective first panel, either bright field image (**A-C**) or the membrane marker WGA (**D-F**). Nuclear pore images were taken with standard fluorescent microscopy (**A-C**). Images for mitochondrial staining are single confocal slices (**D-F**). Scale bars are 10 microns.
(TIF)

S2 Fig Hemocytes in *Drosophila phalerata* examined with scanning electron microscope. Additional imaging of *D*. *phalerata* with SEM, including plasmatocytes (**A**), and type-II nematocytes (**B** & **C** [10 μm],). Scale bars are 5 microns unless indicated with brackets.
(TIF)

S3 Fig Hemocytes in *Drosophila falleni* examined with scanning electron microscope. Additional imaging of *D*. *falleni* with SEM, including plasmatocytes (**A** [5 μm]), type-I nematocytes (**B** & **C**), and type-II nematocytes (**D, E** [5 μm], & **F**). Scale bars are 10 microns unless indicated with brackets.
(TIF)

S4 Fig Type-I and type-II nematocytes are viable cells containing RNA. Hemocytes from *D*. *falleni* and *D*. *phalerata* were examined for cell viability and nucleic acid staining with propidium iodide. After fixation type-I (**A** & **E**) and type-II (**B** & **F**) nematocytes are positive for the nucleic acid dye (propidium iodide), although only the type-I nematocytes are positive for DAPI signal. Live cells, both type-I (**C** & **G**) and type-II (**D** & **H**) nematocytes do not have propidium iodide staining, indicating cell viability. Images are taken with standard fluorescent microscopy and scale bars are 10 microns.
(TIF)

S5 Fig Cellular structure and organelle composition of *Drosophila falleni* hemocytes visualized with transmission electron microscopy. Ultrastructure and organelles of three hemocyte classes were captured; key features include nucleus (N), nuclear envelope (carrot), microtubules (T), and mitochondria (M). Based on morphology, imaged cells are classified as plasmatocytes (**A** [2 μm]) and corresponding magnification (**B** [500 nm]), a type-I nematocyte (**C** [2 μm]) and magnification (**D** [500 nm]), as determined by the presence of a nucleus and irregular cell body shape. Finally, type-II nematocytes are shown, and clearly possess long microtubules and mitochondria (**E** [2 μm] & **G** [1 μm]), respective magnifications are also shown (**F** [500 nm] & **H** [500 nm]). Scale bar lengths are indicated with brackets.
(TIF)

S6 Fig Cellular structure and organelle composition of *Drosophila phalerata* hemocytes visualized with transmission electron microscopy, additional images. Ultrastructure and organelles hemocytes were captured; key features include nucleus (N), nuclear envelope (carrot), microtubules (T), and mitochondria (M). Based on morphology, imaged cells are classified as plasmatocytes (**A** [1 μm]) and a type-II nematocyte (**B** [2 μm]) and magnification (**C** [500 nm]). An image of a suspected multi-cellular structure was captured (**D** [1 μm]) and corresponding magnification (**E** [500 nm]). Finally, type-II nematocyte is shown with long microtubules and abundance of mitochondria (**F** [1 μm]), inset shows the entire cell. Scale bar lengths are indicated with brackets.
(TIF)

S7 Fig Type-I and type-II nematocytes have extensive microtubule networks. Tubulin, visualized with anti-tubulin, is seen in type-I (**A** & **C**) and type-II (**B** & **D**) nematocytes. DNA is counterstained with DAPI. Images are maximum projections from confocal microscope. Scale bars are 10 microns.
(TIF)

S8 Fig Nematocytes do not exhibit phagocytic activity. Representative images from the phagocytosis assay. A plasmatocyte, the traditional phagocytic cell in drosophila is shown with a fluorescent bead (**A**). The nematocytes, although neighboring florescent beads, does not have any within its cell body (**B**). Images are from standard fluorescent microscopy and phase contrast, scale bars are 10 microns.
(TIF)

S9 Fig Growth and extension of type-I nematocyte spindle appendages is microtubule dependent. Live cell phase contrast imaging captures a type-I nematocyte. Over time this cell is observed growing and extending cell projections (**A**). Time lapse is in 3 minute intervals. The camera position is adjusted at T24 to capture cell extension. Type-I nematocytes appear to require dynamic microtubules for proper spindle morphology. Hemolymph treated with colchicine had a greatly reduced number of type-I nematocytes with spindle projections, required for multi-cellular structures. Asterisk indicates p-value of 2.2e-16 from a Fishers exact test.
(TIF)

S1 Media File Time-lapse movie of type-II nematocyte dynamic movement. Time-lapse imaging from Fig 5 is displayed in movie form to ease visualization of cell movement. Images are taken at two minute intervals; the movie file is sped up to one frame per second.
(MP4)

S2 Media File Time-lapse movie of the formation of a type-II nematocyte. Time-lapse imaging from Fig 6 is displayed in movie form to ease visualization of cell movement. Images are taken at two-minute intervals; the movie file is sped up to one frame per second. White carrot indicates specific region of interest.
(MP4)

S3 Media File Time-lapse movie of type-II nematocyte dynamic movement and interaction with other cells. Time-lapse imaging shows two type-II nematocytes (T0) extending its appendages. By the final time point this cell has intertwined or connected to a neighboring cell, forming a multicellular structure. Time intervals are 2 minutes with DIC microscopy and perfect focus; the movie file is sped up to one frame per second.
(MP4)

S1 Table Stock listings and source information. (XLSX)
