# Supplementary figures and images for: Nematocytes: Discovery and characterization of a novel anculeate hemocyte in Drosophila falleni and Drosophila phalerata
Source: PLoS One. 2017 Nov 15;12(11):e0188133. doi: 10.1371/journal.pone.0188133 (PMC5687758; doi:10.1371/journal.pone.0188133)

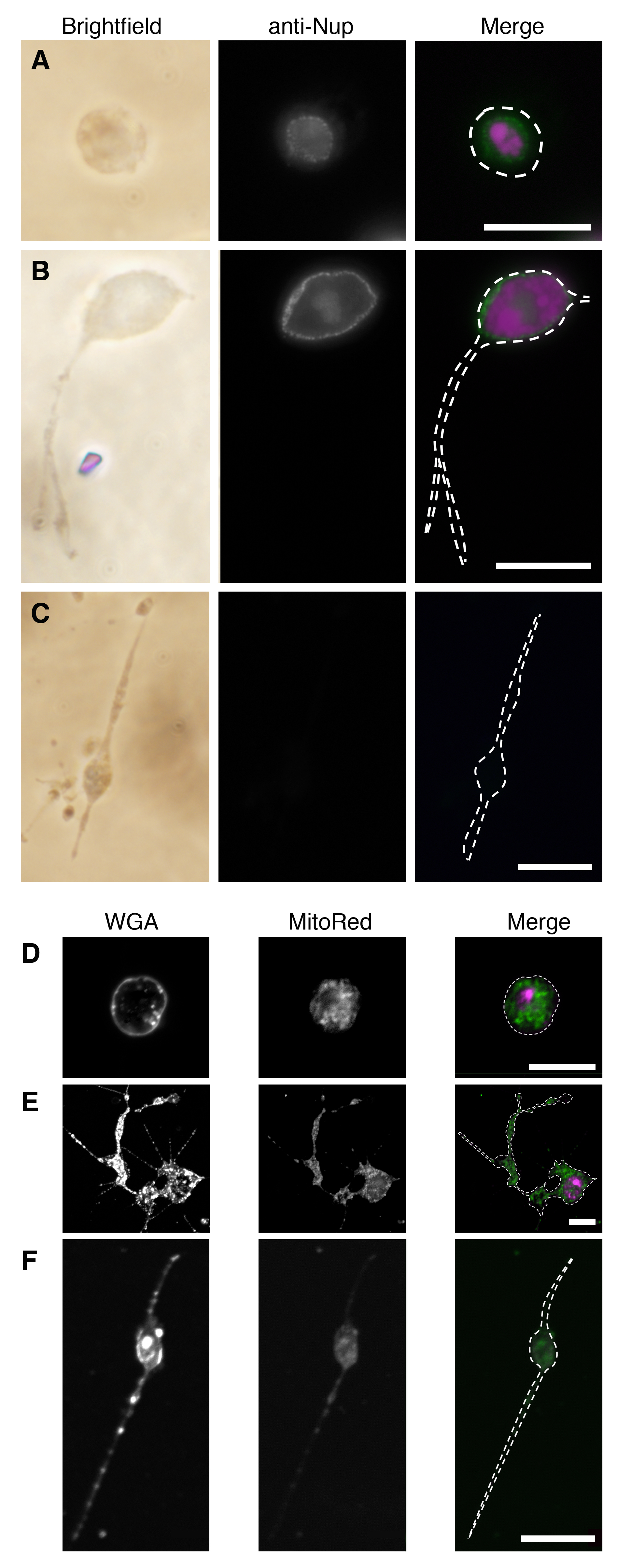

Supplement: S1 Fig — Nuclear pore complexes were observed in both DAPI positive cell types; plasmatocytes (A) and type-II nematocytes (B), as detected by immunofluorescence. Type-II nematocytes are negative for both DAPI and nuclear pore complex signal, making them distinct from the other two hemocytes (C). Mitochondria were detected using MitoTracker-Red and pseudo colored green. Each of the cell types; plasmatocytes (D), type-I nematocytes (E), and type-II nematocytes (F) are positive for this stain. DAPI is shown in magenta within the merged panels and the cell outline (white dashed line) is based on the respective first panel, either bright field image (A-C) or the membrane marker WGA (D-F). Nuclear pore images were taken with standard fluorescent microscopy (A-C). Images for mitochondrial staining are single confocal slices (D-F). Scale bars are 10 microns. (TIF) [file pone.0188133.s001.tif]

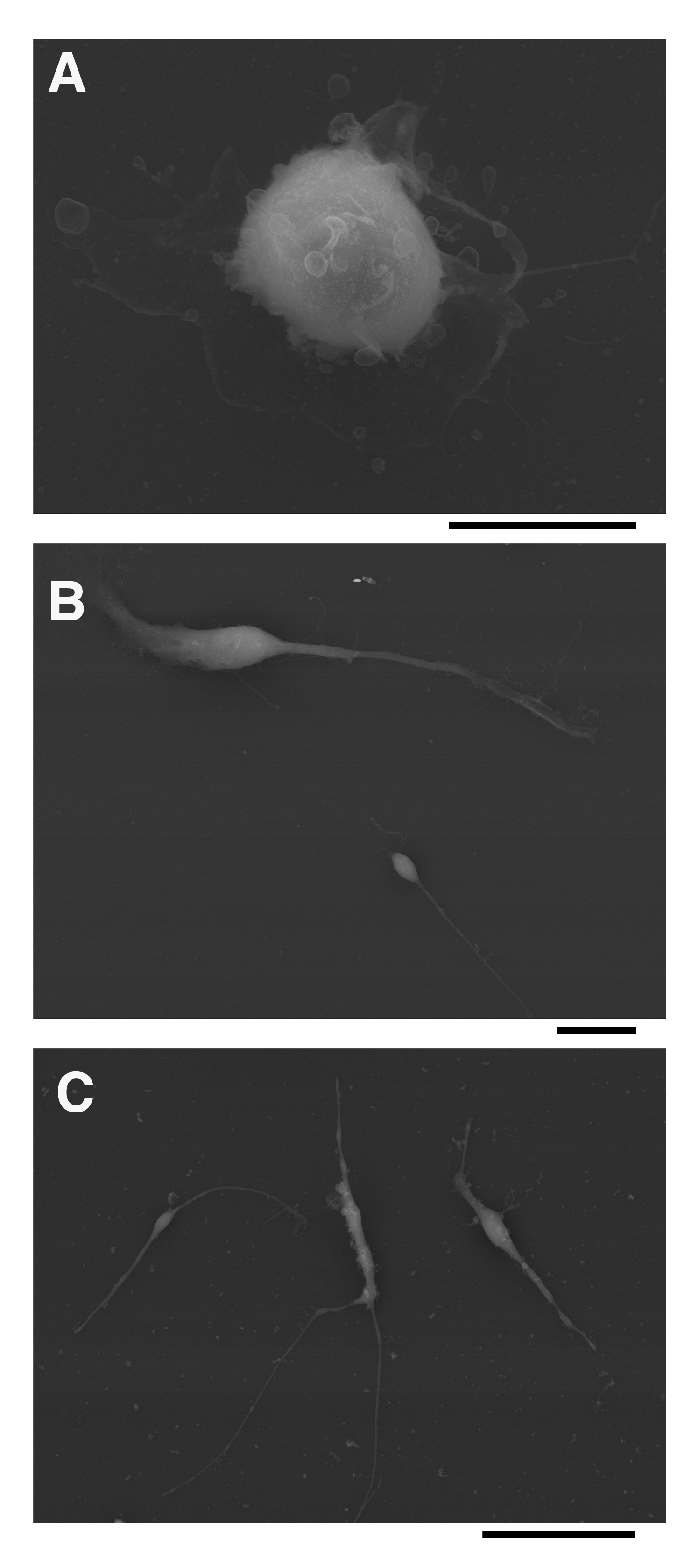

Supplement: S2 Fig — Additional imaging of D. phalerata with SEM, including plasmatocytes (A), and type-II nematocytes (B & C [10 μm],). Scale bars are 5 microns unless indicated with brackets. (TIF) [file pone.0188133.s002.tif]

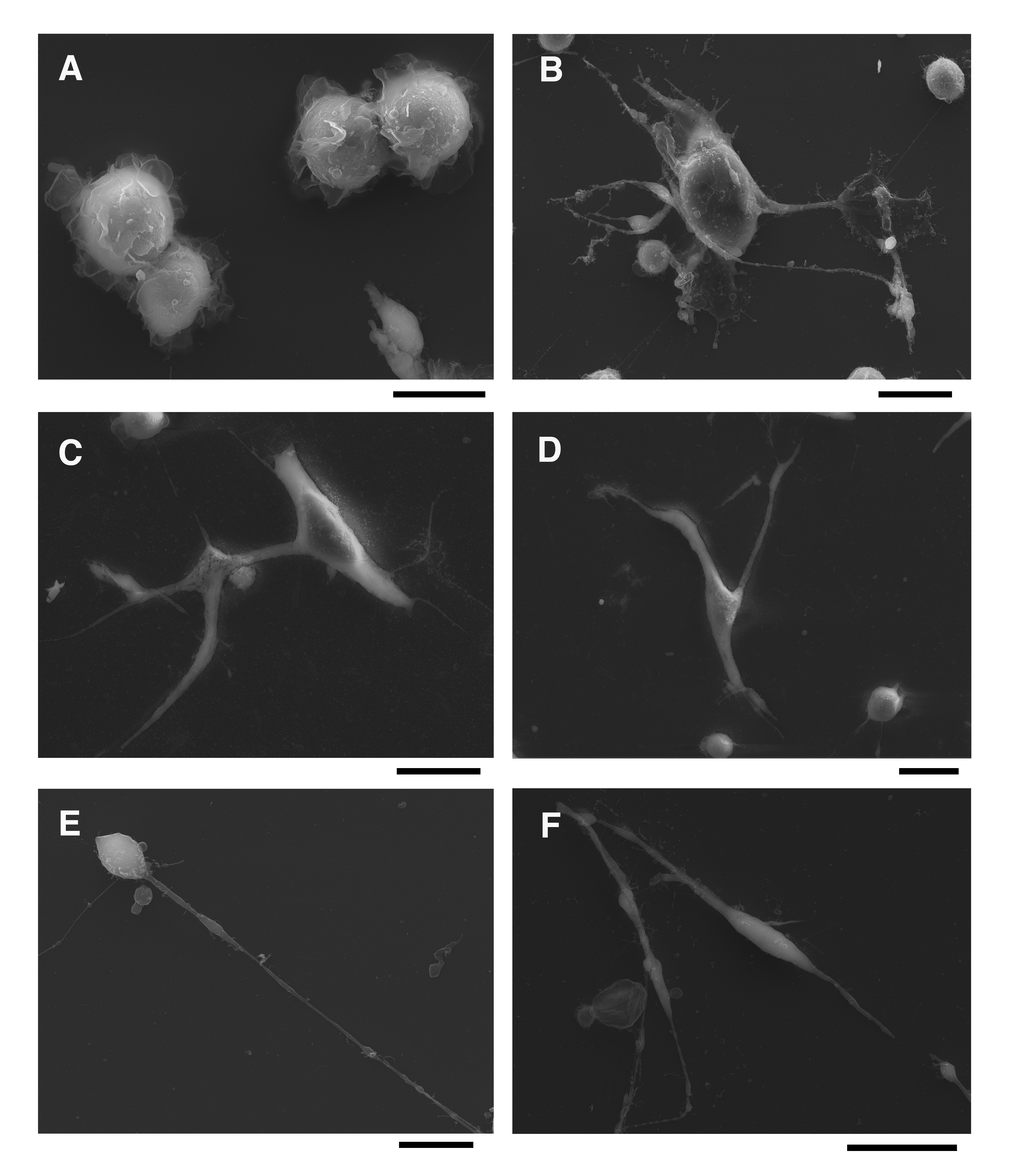

Supplement: S3 Fig — Additional imaging of D. falleni with SEM, including plasmatocytes (A [5 μm]), type-I nematocytes (B & C), and type-II nematocytes (D, E [5 μm], & F). Scale bars are 10 microns unless indicated with brackets. (TIF) [file pone.0188133.s003.tif]

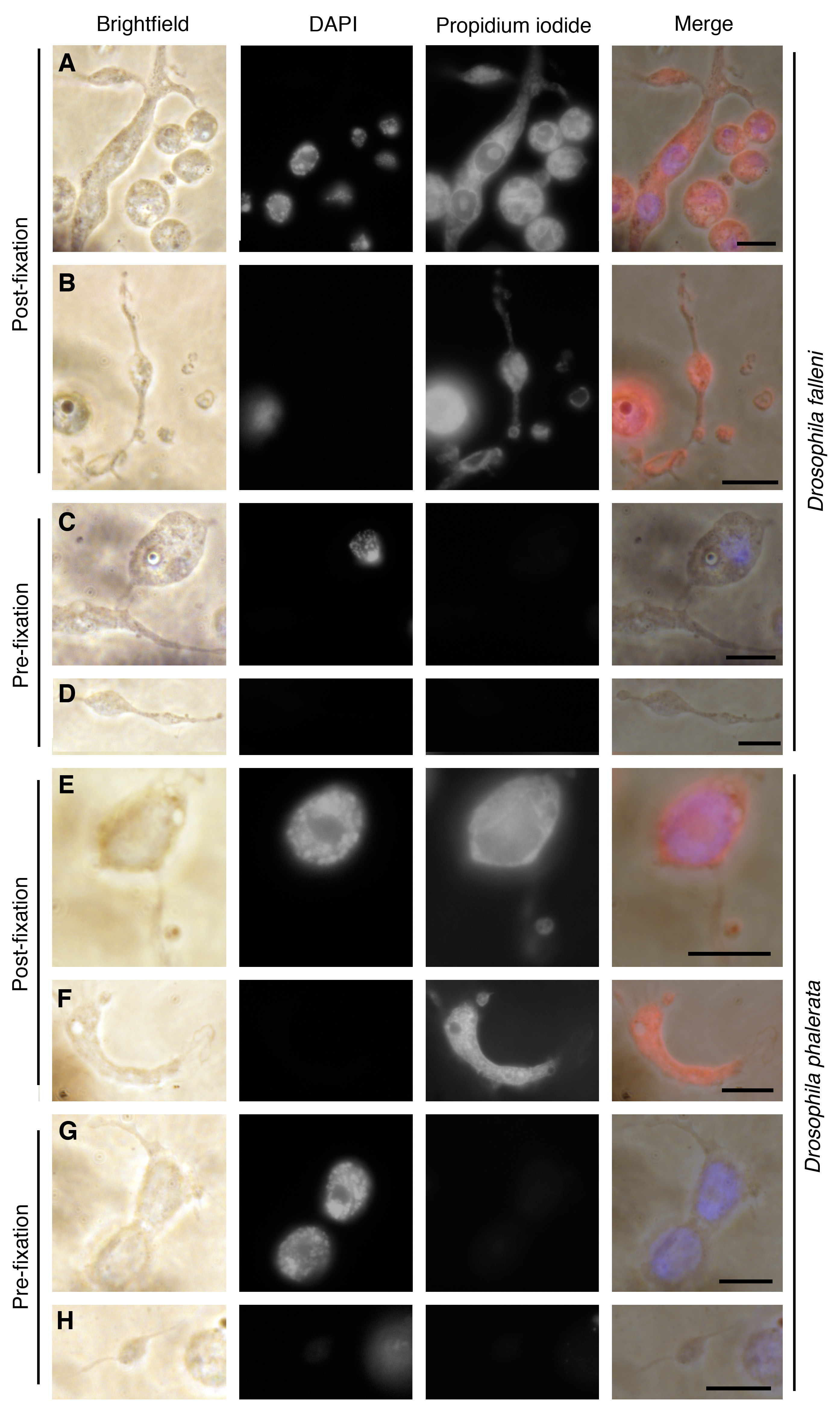

Supplement: S4 Fig — Hemocytes from D. falleni and D. phalerata were examined for cell viability and nucleic acid staining with propidium iodide. After fixation type-I (A & E) and type-II (B & F) nematocytes are positive for the nucleic acid dye (propidium iodide), although only the type-I nematocytes are positive for DAPI signal. Live cells, both type-I (C & G) and type-II (D & H) nematocytes do not have propidium iodide staining, indicating cell viability. Images are taken with standard fluorescent microscopy and scale bars are 10 microns. (TIF) [file pone.0188133.s004.tif]

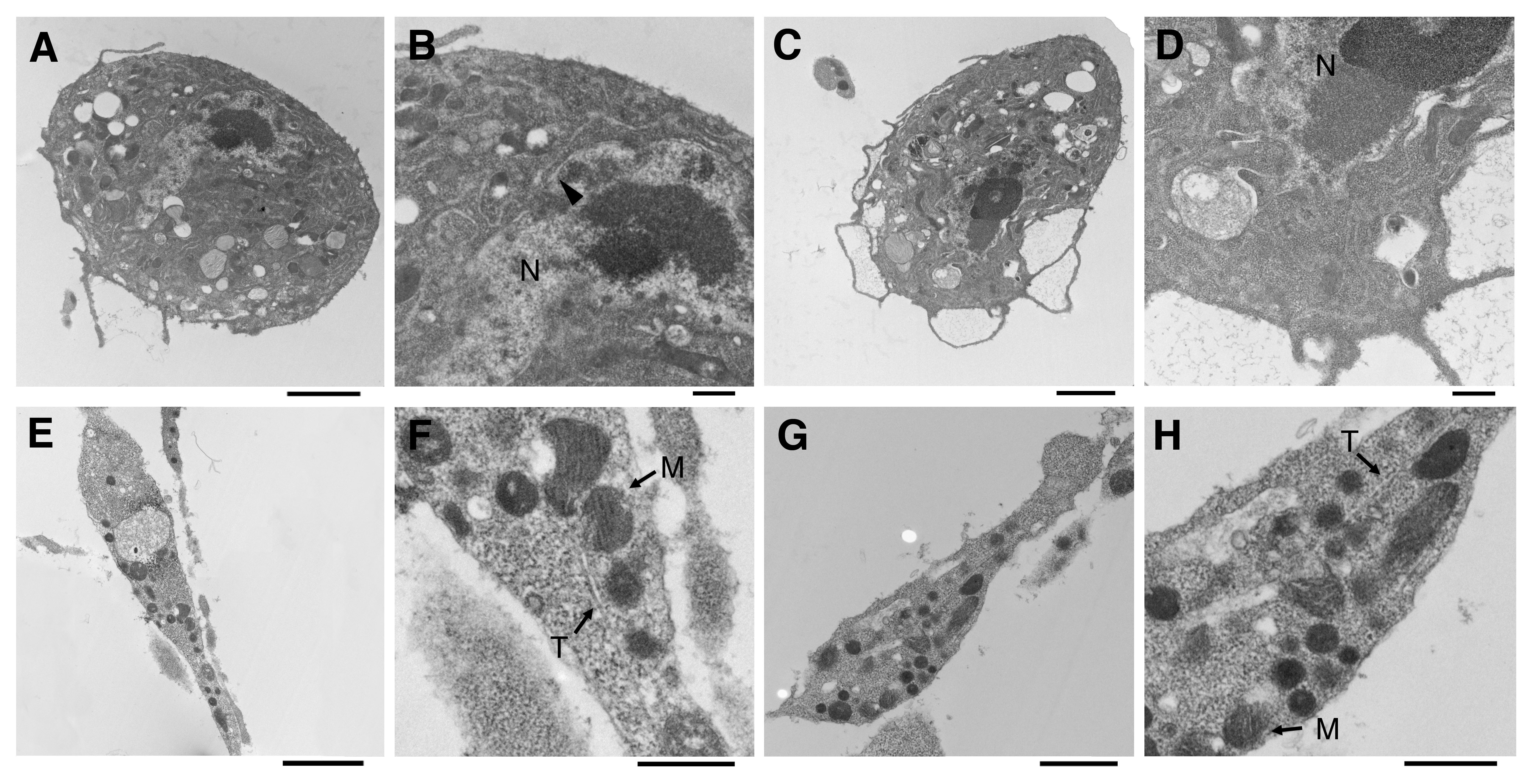

Supplement: S5 Fig — Ultrastructure and organelles of three hemocyte classes were captured; key features include nucleus (N), nuclear envelope (carrot), microtubules (T), and mitochondria (M). Based on morphology, imaged cells are classified as plasmatocytes (A [2 μm]) and corresponding magnification (B [500 nm]), a type-I nematocyte (C [2 μm]) and magnification (D [500 nm]), as determined by the presence of a nucleus and irregular cell body shape. Finally, type-II nematocytes are shown, and clearly possess long microtubules and mitochondria (E [2 μm] & G [1 μm]), respective magnifications are also shown (F [500 nm] & H [500 nm]). Scale bar lengths are indicated with brackets. (TIF) [file pone.0188133.s005.tif]

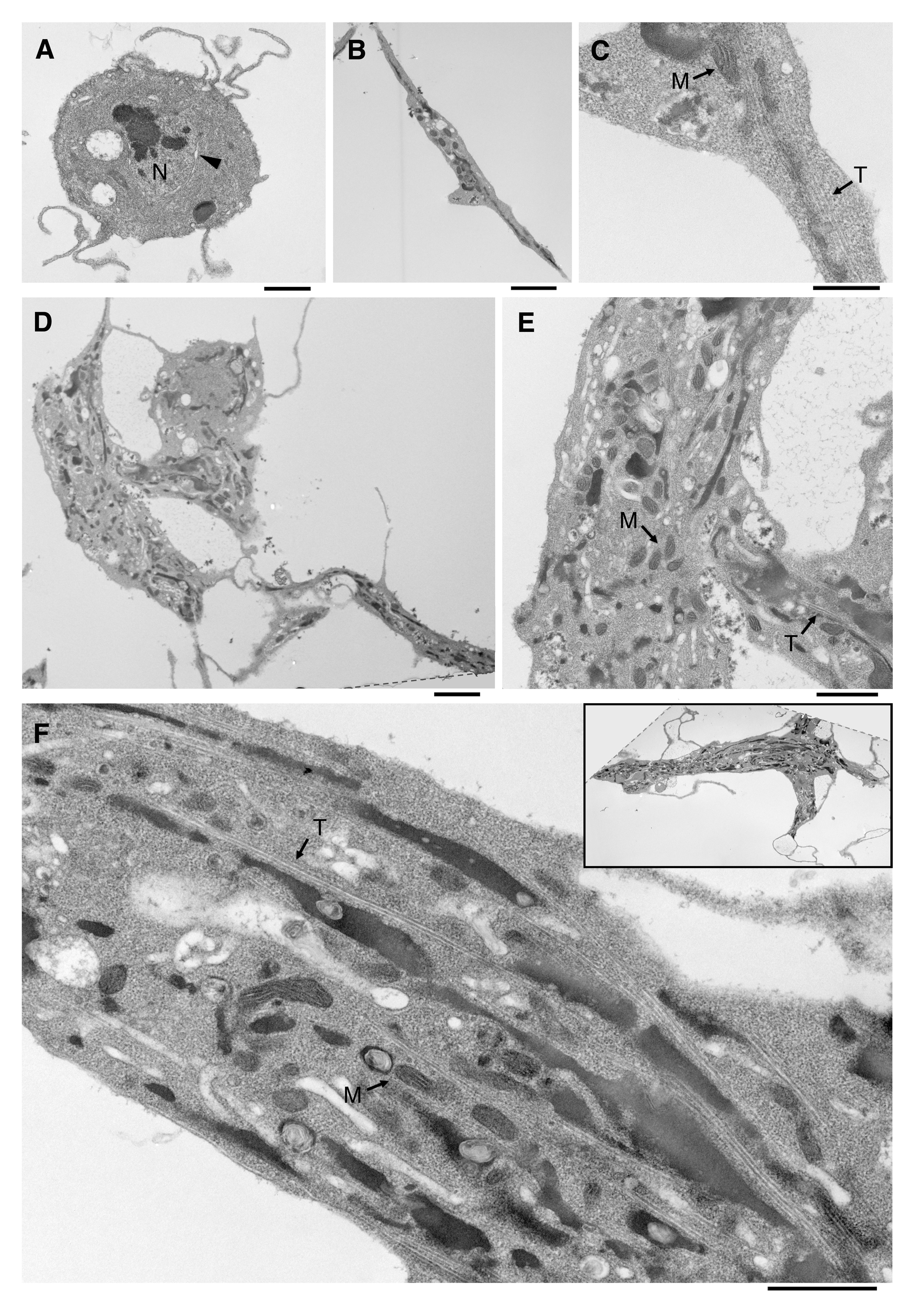

Supplement: S6 Fig — Ultrastructure and organelles hemocytes were captured; key features include nucleus (N), nuclear envelope (carrot), microtubules (T), and mitochondria (M). Based on morphology, imaged cells are classified as plasmatocytes (A [1 μm]) and a type-II nematocyte (B [2 μm]) and magnification (C [500 nm]). An image of a suspected multi-cellular structure was captured (D [1 μm]) and corresponding magnification (E [500 nm]). Finally, type-II nematocyte is shown with long microtubules and abundance of mitochondria (F [1 μm]), inset shows the entire cell. Scale bar lengths are indicated with brackets. (TIF) [file pone.0188133.s006.tif]

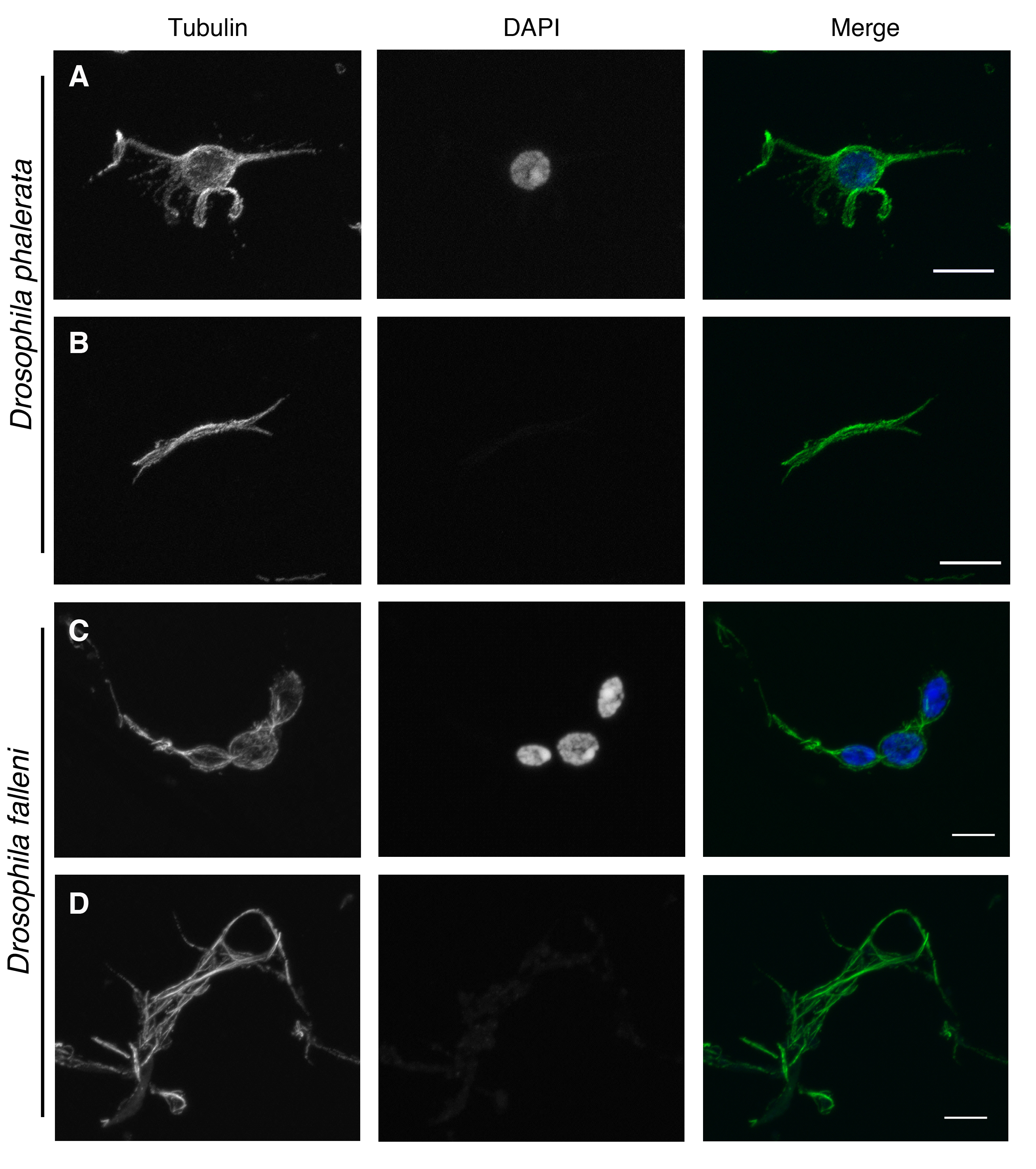

Supplement: S7 Fig — Tubulin, visualized with anti-tubulin, is seen in type-I (A & C) and type-II (B & D) nematocytes. DNA is counterstained with DAPI. Images are maximum projections from confocal microscope. Scale bars are 10 microns. (TIF) [file pone.0188133.s007.tif]

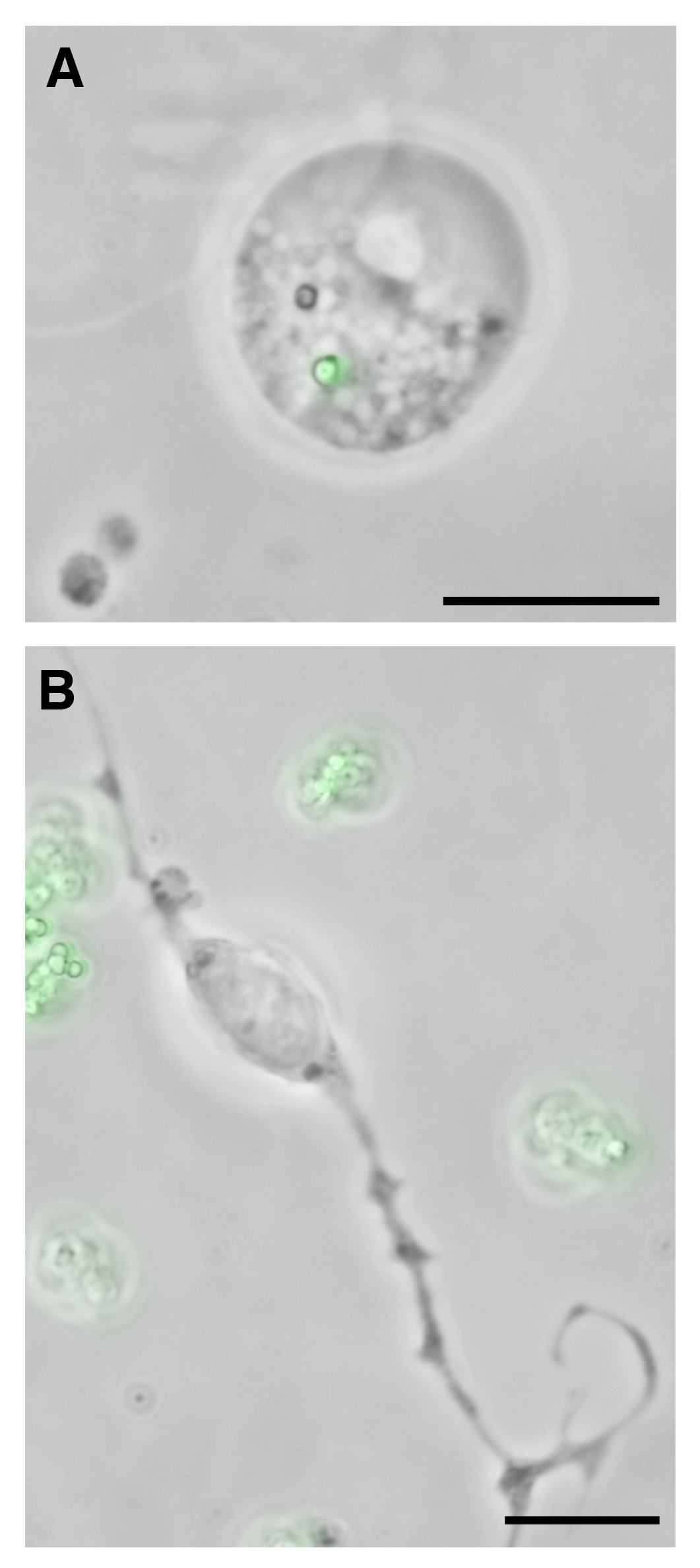

Supplement: S8 Fig — Representative images from the phagocytosis assay. A plasmatocyte, the traditional phagocytic cell in drosophila is shown with a fluorescent bead (A). The nematocytes, although neighboring florescent beads, does not have any within its cell body (B). Images are from standard fluorescent microscopy and phase contrast, scale bars are 10 microns. (TIF) [file pone.0188133.s008.tif]

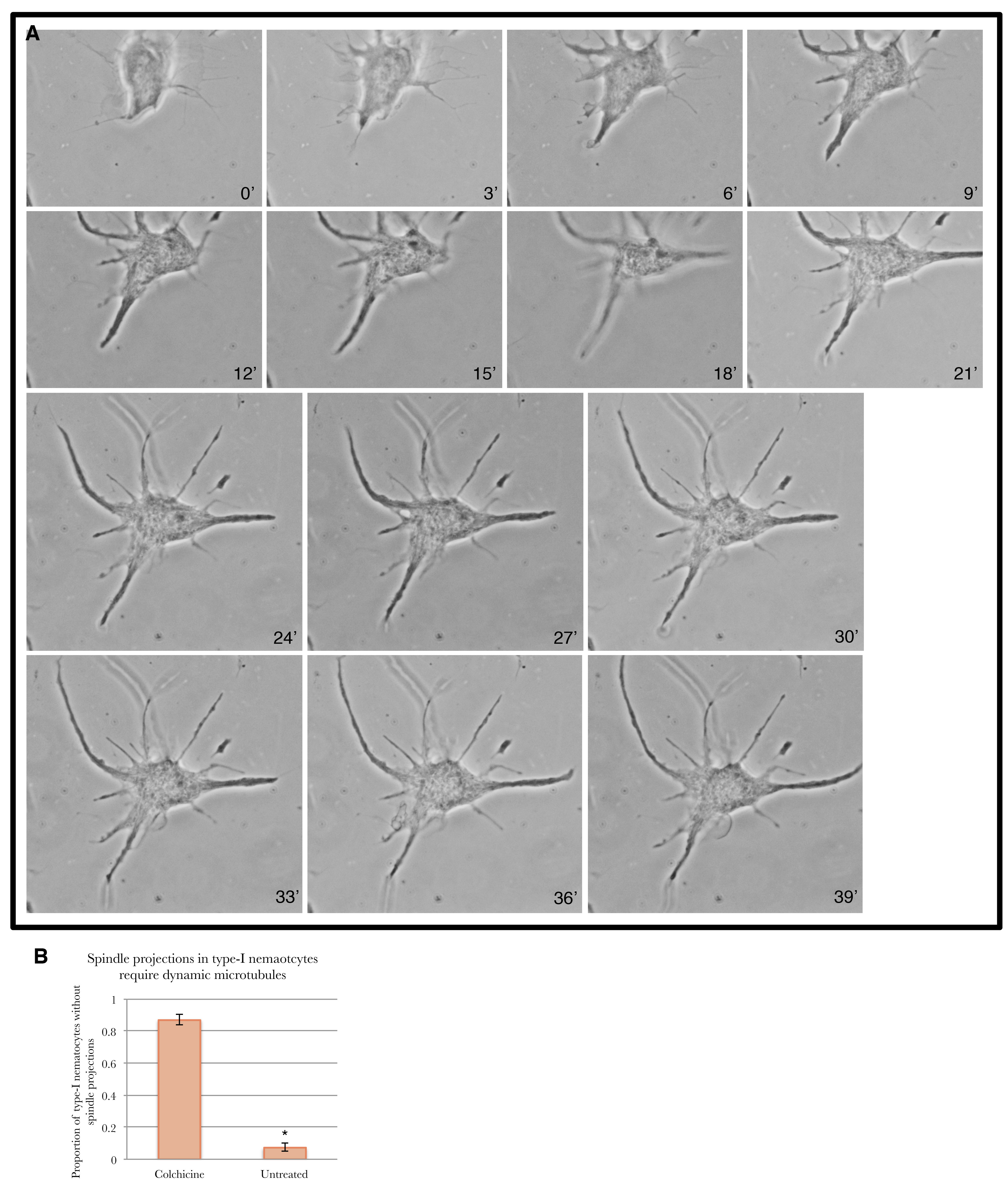

Supplement: S9 Fig — Live cell phase contrast imaging captures a type-I nematocyte. Over time this cell is observed growing and extending cell projections (A). Time lapse is in 3 minute intervals. The camera position is adjusted at T24 to capture cell extension. Type-I nematocytes appear to require dynamic microtubules for proper spindle morphology. Hemolymph treated with colchicine had a greatly reduced number of type-I nematocytes with spindle projections, required for multi-cellular structures. Asterisk indicates p-value of 2.2e-16 from a Fishers exact test. (TIF) [file pone.0188133.s009.tif]
